# Organic electrochemical transistors printed from degradable materials as disposable biochemical sensors

**DOI:** 10.1038/s41598-023-38308-1

**Published:** 2023-07-15

**Authors:** Nicolas Fumeaux, Claudio Pinto Almeida, Silvia Demuru, Danick Briand

**Affiliations:** Soft Transducers Laboratory, Ecole Polytechnique Fédérale de Lausanne (EPFL), Rue de la Maladière 71b, CH-2000 Neuchâtel, Switzerland

**Keywords:** Sensors and biosensors, Electrochemistry, Design, synthesis and processing

## Abstract

Transient electronics hold promise in reducing electronic waste, especially in applications that require only a limited lifetime. While various degradable electronic and physical sensing devices have been proposed, there is growing interest in the development of degradable biochemical sensors. In this work, we present the development of an organic electrochemical transistor (OECT) with degradable electrodes, printed on an eco- and bioresorbable substrate. The influence of the design and materials for the contacts, channel and gate of the transducer, namely poly(3,4-ethylene dioxythiophene):polystyrene sulfonate (PEDOT:PSS) and carbon, is systematically evaluated for the development of OECT-based transient biosensors. The sensing capabilities of the electrochemical transistors are demonstrated with ionic solutions as well as for the enzyme-based detection of glucose. The disposable OECTs show comparable performance to their non-degradable counterparts. Their integration with highly conductive degradable and printable zinc tracks is studied for the realization of interconnects. These eco-friendly OECTs may find applications as disposable and sustainable biochemical sensors, and constitute a step towards bioresorbable biosensors.

## Introduction

In recent years, interest in seamless, low-cost and reliable biosensors has been steadily increasing, targeting applications in point-of-care testing^1,2^, continuous monitoring through wearables^3,4^ or implanted biosensors^5^. In this context, organic electrochemical transistors (OECTs) have emerged as a promising alternative to potentiometric, amperometric or ion-sensitive field-effect transistors (ISFET)-based sensors^6^. Recent interest in OECTs has been motivated by their compatibility with mechanically compliant substrates^7^, high transconductance^8^, reference-free operation^9^ and simplified microfluidic integration^10^. Precise monitoring of various analytes has been demonstrated leveraging the high signal amplification properties of OECTs. Examples include the sensing of pH^11^, electrolytes such as sodium and potassium^12^, metabolites including glucose and lactate^13^ or neurotransmitters such as dopamine or epinephrine^14,15^. OECTs consist of three terminals (source, drain and gate). The source and drain contacts are usually fabricated with metals such as gold or silver and the channel consists of a conductive polymer layer connecting the source and the drain. The channel is most often based on poly(3,4-ethylene dioxythiophene):polystyrene sulfonate (PEDOT:PSS)^16^, although other materials such as polypyrrole have been explored^8^. A variety of materials has been used to fabricate the gate electrode, although silver/silver chloride or platinum are the most common choices for gate fabrication^8^. The gate and channel are in contact with an electrolyte (in liquid or gel form) and cations from the electrolyte diffuse into the channel if a positive gate potential is applied, and de-dope the conductive polymer by compensating the anions of the PSS dopant. This relatively simple architecture is compatible with planar designs and high-density integration into microfluidic systems or complex organic electronic circuits^17^.

Advantageously, the fabrication of OECTs, in particular of the conductive channel, is compatible with solution-based fabrication methods and additive manufacturing, enabling cost-efficient manufacturing and rapid prototyping on flexible substrates^10^. This opens new possibilities in terms of the combination of materials that can be used in the manufacturing of OECTs, in particular the use of degradable materials. Degradable electronics refer to electronic systems and components that can degrade in an environment of interest spontaneously, in a controlled amount of time, and without releasing byproducts that are harmful to that environment^18^. With concerning amounts of electronic waste being generated, as well as exploding numbers of connected Internet of Things (IoT) devices^19^, there is growing interest in transient electronic systems with a service life of a few days to a few months. Although advances have been made in the manufacturing of fully degradable functional devices, i.e. antennas^20^, batteries^21^ and physical as well as environmental sensors^22,23^, investigations into degradable biosensors remain relatively limited^24^.

Advances have been made in proposing new materials for the OECT terminals, in particular the gate electrode, as its properties play a key role in modulating the transistor’s behavior. While Ag/AgCl gates offer the advantage of being non-polarizable, Au gates present little electrochemical activity in the range of voltages typical for OECT-based biosensing. Au and PEDOT:PSS gates have been explored for OECT-based biosensors, with the advantage of expanding the possibilities for bio-functionalizing the gate electrode^6,25^. PEDOT:PSS gates and contacts have been investigated, simplifying notably their manufacturing^26^. An all-PEDOT:PSS OECT was presented and shown to measure dopamine concentrations reliably and specifically^27^. Various forms of carbon have also been investigated for the realization of gate electrodes for OECTs^8^. Activated carbon gates, for example, showed increased drain current modulation due to the large specific surface area of the carbon material^9^. Recently, screen-printed carbon-gated OECTs were shown to be suitable for the detection of uric acid after functionalization of the carbon gate with platinum and Uricase^28^. Transient or recyclable materials such as paper^26^ have been proposed as substrates for OECTs. Polylactic acid (PLA)^24^ and Poly(lactic-co-glycolic acid)^29^ (PLGA) have been studied as degradable substrates for OECTs^30^, as well as diacetate cellulose^31^. These studies, however, relied on non-degradable contacts for the operation of the printed OECTs. More recently, Khan et al.^32^ proposed a fully printed OECT on cellulose acetate (CA) for the selective detection of glucose. The OECT is made of degradable materials and CA is a biocompatible material that is suitable as a substrate for transient biosensors.

In this work, we present disposable and biocompatible OECTs based on carbon, PEDOT:PSS and PLA as substrate. Challenges in the fabrication of transient electronic devices come from the low-temperature tolerance^18^ of biopolymeric substrates and reaching adhesion of the PEDOT:PSS channel material on the biopolymer^33^, which is often deposited from an aqueous solution. A fully additive fabrication process is developed to address these challenges, leveraging screen and inkjet printing. The influence of the gate material choice, as well as the gate geometry, are studied, and these parameters are optimized for the fabrication of transient OECTs for ions and metabolite sensing. The transistor characteristics of the devices as well as their sensing behavior and reproducibility are characterized. Finally, the degradable OECTs are integrated with highly conductive transient zinc metal traces, which are of interest for interconnection with other degradable electronic circuits and could allow, for example, the wireless operation of the biochemical chemical sensors^34^.

## Results and discussion

### OECTs design and materials

The disposable electrochemical transistors presented in this work are entirely fabricated with environmentally benign and biocompatible materials. The materials used in these devices are amenable to the manufacturing of transient biosensors. Carbon and PEDOT:PSS were chosen in order to respect the aforementioned criterion, and the specific materials combinations are optimized here for two applications: ion sensing and glucose detection. These two analytes rely on different sensing mechanisms, namely diffusion of cations into the conducting channel versus glucose oxidation reactions generating hydrogen peroxide and catalyzed by glucose oxidase^6^.

**Figure 1:**
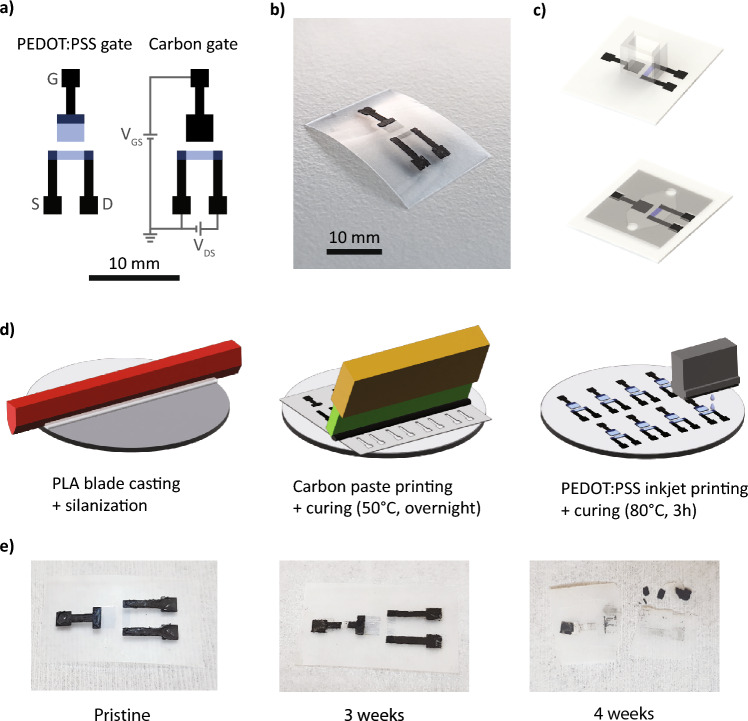
Design and fabrication of the degradable OECTs. (**a**) Layout of the two OECT designs (PEDOT:PSS-gated and carbon-gated). (**b**) Picture of a PEDOT:PSS-gated device. (**c**) Recording setups used in the experiments: PMMA reservoir (top) and microchannel (bottom). (**d**) Fabrication process for the additive manufacturing of degradable OECTs: (i) PLA substrate casting and surface modification, (ii) carbon paste stencil printing and curing and (iii) inkjet printing of PEDOT:PSS channel and curing. (**e**) Degradation of a transient device in soil at different timepoints.

Figure 1a shows the configuration of the carbon-gated and PEDOT:PSS devices, as described above, as well as the contacts (gate, source and drain) and the voltages that mediate the transistor behavior. A PEDOT:PSS-gated device is shown on the left and a device with a carbon gate on the right. The channel has an effective length of 3 mm and a width of 1 mm, resulting in a W/L ratio of 1/3. The gate geometry, in particular the area ratio between the gate and the channel, was varied. The contacts and channel are arranged in a planar configuration, as this allows for easier microfluidic integration. An optical image of such a device, in this case with a PEDOT:PSS gate electrode is shown in Fig. 1b. Based on the measurement to be performed, the test solution was contained either in a polymethylmethacrylate (PMMA) reservoir or a microfluidic channel (Fig. 1c), which was fabricated from laser-cut polydimethylsiloxane (PDMS) and polyethylene terephthalate (PET).

As mentioned above, the materials considered in this work to fabricate the transistors are carbon and PEDOT:PSS. Carbon inks have been shown to be degradable in a standard compost^35^ and considered to be compatible with implanted use^36^. Moreover, carbon and carbon allotropes in general are of interest for enzymatic detection of molecules due to their high surface area and electron transfer rate^37^. PEDOT:PSS is a biocompatible material^30^, has been observed to break-down in moist environments^38^, and is considered environmentally benign^39^. The contacts of the OECTs are formed with a screen printable carbon-based paste. The carbon paste is obtained by mixing graphite flakes, carbon black, pentanol and shellac as described in work by Poulin et al.^40^. This formulation was chosen for the water insolubility of the shellac, allowing the contacts to operate in humid environments. The carbon contacts for the source and drain have a resistance of 44.2 ± 5.3 Ω. The channel is fabricated by inkjet printing of a water-based PEDOT:PSS solution, as well as the gate in some configurations. The conductive polymer solution is modified with dimethyl sulfoxide (DMSO), which has been shown to increase PEDOT:PSS conductivity by enhancing PEDOT:PSS cohesion and the creation of PEDOT:PSS-rich domains^41,42^, as well as favor stable printing due to its low volatility^43^. The inkjet-printed PEDOT:PSS channel has a thickness of 0.50 ± 0.05 µm and a resistance of 1.54 ± 0.06 kΩ (n = 3). The gate electrode is constituted of a carbon contact, which is extended as a gate in some experiments or is in contact with a PEDOT:PSS gate in a second configuration, as seen in Fig. 1a. The fabrication process flow is shown in Fig. 1d. The degradation of the devices is qualitatively assessed with a degradation test in soil at 55 °C (Fig. 1e), as previously used for assessing the degradation of PLA substrates^44^. The devices do not undergo visible physical degradation until the third week, when PLA is observed to become opaque and crack, and PEDOT:PSS to break down and fragment. After a month of degradation, the PLA substrate as well as the carbon contacts have separated in smaller pieces and the PEDOT:PSS channel is almost entirely broken down.

### Influence of gate material and geometry

**Figure 2:**
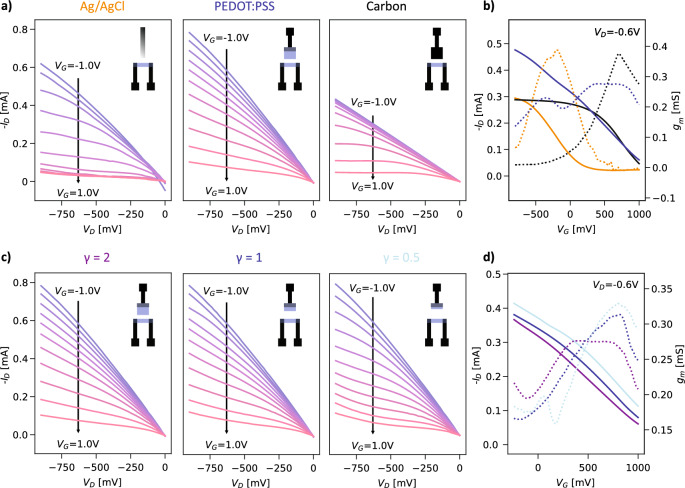
Influence of the gate material and dimensions on the behavior of the transistors. (**a**) Output curves for Ag/AgCl, PEDOT:PSS and carbon gates. (**b**) Corresponding transfer curves (solid lines) and transconductance (dotted lines) for the gate materials. (**c**) Output curves for different gate area to channel area ratios: (left) larger gate, ɣ = 2, (middle) medium gate, ɣ = 1, (right) smaller gate, ɣ = 0.5. (**d**) Corresponding transfer curves (solid lines) and transconductance (dotted lines) for the gate-to-channel ratios.

Degradable materials, unsurprisingly, often exhibit higher reactivity in aqueous solutions^18^, and thus, we look at the influence of carbon and PEDOT:PSS as gate material on the electrical characteristics of the transistors. This was first evaluated on an inert polyimide (PI) substrate to avoid potential influence from the biodegradable substrate on the OECTs behavior when in an aqueous solution. Indeed, as can be seen in Fig. 2a on the left, an OECT with carbon contacts and Ag/AgCl gate electrode displays a very similar behavior as OECTs with the same geometry and channel characteristics seen in the literature^8^. The transconductance maximum is observed at a V_gs_ of approximately  − 200 mV and a transconductance of 0.389 mS is obtained as seen in Fig. 2b, showing the drain current as a function of the gate voltage as well as the transconductance. The transconductance peak for Ag/AgCl gated OECTs is usually found at gate voltages around 0 V, however negative values of a few hundreds of millivolts have been observed in the literature^12,45^, in particular when the resistance of the contact was negligible with respect to the resistance of the channel^46^. An explanation for this behavior has been proposed by Paudel et al.^47^. With lower (more negative) source-drain voltages (V_ds_ is − 600 mV in our case), more cations accumulate at the drain electrode, partially de-doping the channel and causing transistor pinch-off at lower (more negative) gate voltages.

The surface properties of the gate electrode are of importance in the transistor behavior achieved. Indeed, contrary to silver/silver chloride (Ag/AgCl) electrodes, which are non-polarizable and operate in a Faradaic regime, polarizable materials such as the ones under consideration here will cause the gate to operate in a capacitive regime^45^. As a consequence, the specific surface of the gate, which has an influence on the double-layer capacitance between the gate and the solution, will affect the transistor behavior^48^. The behavior of the transistors was assessed by measuring their output (drain current *I*_ds_–drain voltage *V*_ds_) and transfer characteristics (drain current *I*_ds_–gate voltage *V*_gs_) as well as calculating their transconductance (*g*_m_ = δ*I*_ds_/δ*V*_gs_). We first look at the influence of the gate material for a fixed geometry and compare it with devices where an Ag/AgCl gate is used. When changing from a non-polarizable Ag/AgCl gate to a polarizable PEDOT:PSS gate, the de-doping behavior is less evident than for an Ag/AgCl gate and happens at higher gate voltages (~ 400 mV, 0.276 mS), in agreement with previous investigations^9,45^. With the carbon contact as the gate material, a similar behavior to the PEDOT:PSS gate is observed, with a transconductance peak at 700 mV and which reaches a value of 0.376 mS. Although the capacitance of the carbon gates is larger than that of the PEDOT:PSS gates (see Fig. S1), the carbon-gated OECT does not show markedly more efficient modulation and only slightly higher transconductance. This could be due to the capacitance of the system being dominated by the channel^16^, as these measurements were conducted with large gates, as well as batch-to-batch variations in the fabrication.

As a further step, we looked at the effect of the gate geometry, as the ratio between the gate and channel areas influences the transistor behavior^49^. For these experiments, we focused on PEDOT:PSS-gated OECTs and varied the aforementioned area ratio (ɣ = A_gate_/A_channel_) between 0.5 and 2, and the results are shown in Fig. 2c,d. As seen in the drawings of the designs in Fig. 2c, the distance between the gate and the channel also changed, however it has been shown in the literature not to affect the electrical behavior of OECTs^49,50^ or their response dynamics^51^ at the distances concerned here. As can be seen in Fig. 2c, the current modulation behavior is more marked for a larger gate relative to the channel (ɣ = 2). Importantly, a peak in transconductance is reached for lower voltages when a large area ratio is used (Fig. 2d), as observed in previous research^52^. This is due to the increased capacitance of the gate with increased area, which reduces the voltage loss at the gate, causing a more efficient modulation of the channel current^45^. Therefore this design (ɣ = 2) was chosen for further experiments in order to favor the operation of the transistors at lower voltages and be farther from the voltage threshold at which water electrolysis occurs.

### Integration on a biodegradable substrate

**Figure 3:**
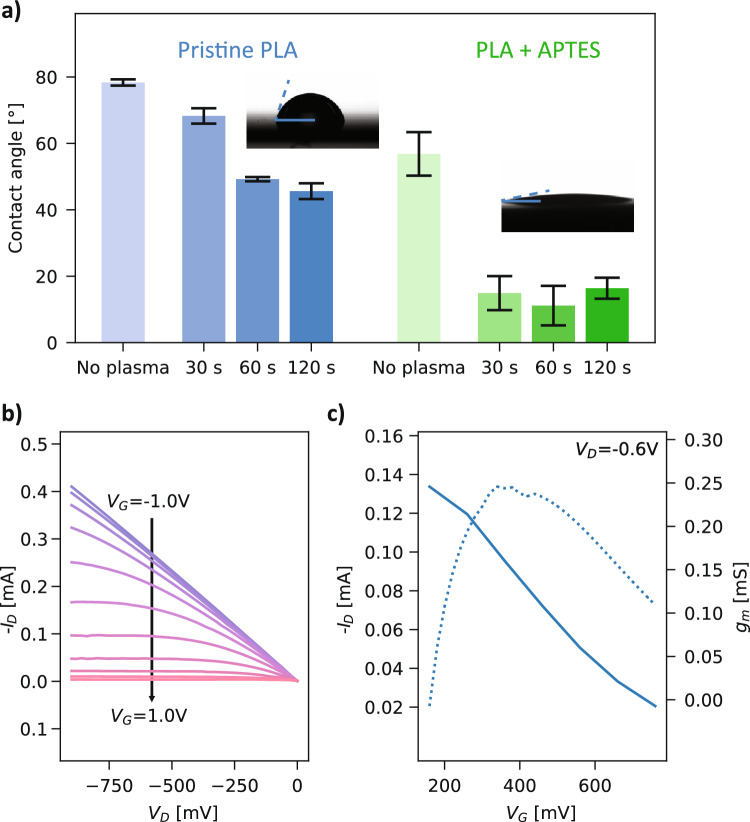
Surface modification of PLA to enable to fabrication of fully degradable OECTs. (**a**) Contact angle of DI water on PLA, with and without silanization and as a function of the time of application of oxygen plasma and in inset: contact angle before (left) and after surface (right) modification (n = 3 for each treatment). (**b**) and (**c**) Electrical characterization of the transient OECT on PLA, respectively output and transfer curves.

With an optimized design for OECTs relying on degradable materials, we transferred their processing on a biodegradable polylactic acid (PLA) substrate. The latter was chosen for its known biodegradability by natural factors, as well as its relative durability in aqueous environments^44^. It effectively strikes a balance between degradability and durability, avoiding for instance a large amount of degradation byproducts immediately fouling the response of the transistor as it comes in contact with the analyte solution. However, its hydrophobicity represents a challenge as the PEDOT:PSS aqueous solution drops deposited by the inkjet printer may not wet the surface, leading to agglomeration and low-quality prints, or delamination of the channel can occur in aqueous media (Supplementary Fig. S1). To circumvent these problems, we study the surface energy of the PLA films and compare it with PI, before and after the use of an oxygen plasma treatment to chemically activate the surface. This was assessed by measuring the contact angle of a droplet of DI water with the substrates and the results are shown in Fig. 3a. The contact angle for polyimide substrates considerably decreases when oxygen plasma treatment is used, from 63.9 ± 5.4° for pristine PI to 14.6 ± 0.7° after 120 s at 40 W (40 kHz), as seen in Supplementary Fig. S2. Conversely, the water contact angle decrease for PLA for the same treatment is less marked, and the substrate remains relatively hydrophobic, with a contact angle of 45.6 ± 2.4° after 120 s of oxygen plasma treatment. To allow for satisfying printing and adhesion of the conductive channel, we functionalized the PLA with 3-aminopropyl)triethoxysilane (APTES), an aminosilane allowing it to obtain a more hydrophilic surface. After APTES spin-coating and subsequent oxygen plasma activation, a contact angle of 16.4 ± 3.2° is achieved. Another challenge with the use of transient substrates such as PLA is the preservation of their integrity during post-processing steps involving heat, especially for extended periods of time. Solution-processed PEDOT:PSS is typically cured at 120 °C^10^ for 20 min or more. We validated that a curing protocol that is compatible with the degradable substrate (80 °C for 3 h) would yield the same transistor behavior as when the standard curing protocol is used. The results presented in Supplementary Fig. S3 show that the longer curing protocol is adequate not only in terms of obtaining a similar channel electrical conductivity, but also transistor behavior for the PEDOT:PSS channel. This allows to fabricate OECTs with fully transient materials, and their output and transfer characteristics are shown in Fig. 3b,c respectively, for a carbon-gated device. A transconductance of 0.247 mS is achieved, which is comparable with OECTs fabricated on non-transient substrates published in recent works^16^. It is interesting to note that our ink formulation contains (3-glycidyloxypropyl)trimethoxysilane, which was originally added to increase to stability of the channel^53^. However, its full cross-linking is only attained when cured at temperatures above 100 °C^54^, and therefore this suggests that the improved adhesion of the PEDOT:PSS to the PLA is largely provided by the aminosilane layer. Finally, the evolution of the transistor behavior of the devices in phosphate-buffered saline (PBS) is evaluated by measuring the transfer behavior repeatedly over approximately one hour, every minute. The results are shown in Fig. S5, and it is observed that the peak transconductance for a PEDOT:PSS-gated device decreases by 20% after 60 measurement cycles. Such a decrease of the current of OECTs has been observed in previous studies and has been inversely linked to the crystallinity of the PEDOT:PSS film^55^.

### Cation sensing experiments

**Figure 4:**
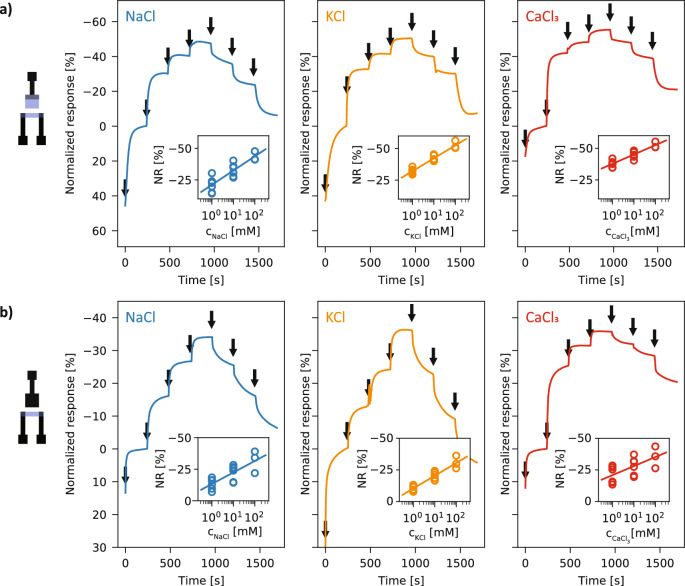
Characterization of the OECTs for ion concentrations measurements. Real-time normalized response to varying concentrations of ions, Na^+^ (left), K^+^ (middle), Ca^3+^ (right) with the injections of the different ion concentrations (DI water, 1, 10, 100, 10, 1 mM, DI water) indicated by arrows, and the regression of the normalized currents in the insets (n = 3), for PEDOT:PSS-gated OECTs (**a**) and carbon-gated OECTs (**b**). The same Y-axis scale was used for all the ions tested.

The degradable OECTs were integrated into a simple microfluidic system to allow for the measurement of decreasing ion concentrations as well as precise control of the ion concentration to which the device is exposed. Prior to measurement, the transistors were flushed with DI water under a gate voltage of 0.6 V for a total of 15 min, adapting a protocol reported in the literature to enhance the stability of PEDOT:PSS-based OECTs^53^. Ionic solutions of NaCl, KCl and CaCl_3_ respectively, in deionized (DI) water were used, in concentrations ranging from 1 to 100 mM. DI water waws injected into the channel via the inlet, followed by increasing concentrations of the test solution every 240 s. Finally, decreasing concentrations of the solution were injected after the same time interval, finishing with a DI water injection. These experiments were conducted with both PEDOT:PSS-gated, in Fig. 4a and carbon-gated OECTs, in Fig. 4b. In the insets, the normalized responses (n = 3) for each ion as well as a logarithmic regression of the responses are shown. The sensitivities for each ion were calculated by extracting the steady-state currents from the raw sensor responses and are reported in normalized terms. Indeed, the resistance of the printed PEDOT:PSS channels, and therefore the magnitude of the source-drain current, may vary between devices. Thus, the response is referenced and normalized with respect to the current I_0_ measured in the device after stabilization in DI water.

Independent devices show similar current modulation and the responses scale linearly with the logarithm of the ion concentration. Carbon-gated and PEDOT:PSS-gated OECTs show qualitatively similar behavior. After the final injection of DI water, the source-drain currents of devices return in the vicinity of the I_0_ current value. However, in order to re-use a device after a set of measurements, we observed that thorough rinsing with DI water for several minutes was necessary to closely restore the initial current. The relative sensitivities of the OECTs comporting a PEDOT:PSS gate are respectively, for the ions Na^+^, K^+^ and Ca^3+^,  − 11.3 ± 1.8%/dec,  − 10.2 ± 0.8%/dec and  − 7.4 ± 1.0%/dec. The carbon-gated OECTs display slightly more variability between devices than the PEDOT: PSS-based devices. This could be due to the printing method for the carbon gate (stencil printing) being less repeatable. For the same ions, the carbon OECTs has the following sensitivities (with standard errors):  − 9.1 ± 2%/dec,  − 10.1 ± 1.1%/dec and  − 7.2 ± 2.4%/dec. Further experiments were conducted with a larger range of concentrations (0.1 mM, 0.5 mM, 1 mM, 5 mM, 10 mM, 50 mM, 100 mM) in order to confirm the sensitivities obtained above for each ion, and the results are reported in Fig. S6. Similar values are obtained for the sensitivities of the devices, with the PEDOT:PSS devices showing slightly higher values, as seen in Fig. 4. Finally, the stabilization of the response of the OECTs to an ion injection was characterized and is shown in Fig. S7. The source-drain current is measured over 20 min after ion injection, repeated three times for the same device. After injection, both the carbon- and PEDOT:PSS-gated sensors’ responses stabilize to reach a plateau. These experiments show that the degradable devices are suitable for the detection of various ions, whether a PEDOT:PSS or carbon gate is used.

### Glucose detection and integration with degradable circuits

**Figure 5:**
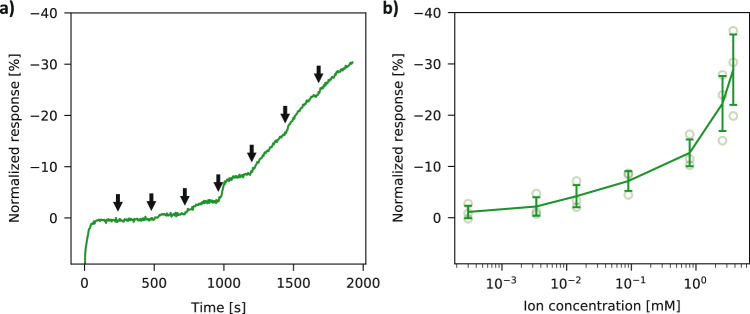
Characterization of the OECTs for the enzymatic detection of glucose. (**a**) Real-time glucose sensing (normalized response current) with increasing glucose concentrations (injections of glucose are indicated by arrows), measured in a PMMA well containing glucose oxidase solution. (**b**) Normalized current response (and standard deviation) versus glucose concentrations (n = 3).

As a further step, the possibility to use these degradable transistors for the detection of glucose was assessed in the proof-of-concept experiments presented hereafter. These biosensors generally rely on the oxidation of glucose by glucose oxidase (GOx) present on or in the vicinity of the sensor, causing an increase in gate voltage^6^. In this work, glucose concentration measurements were conducted in a PMMA well. Degradable carbon-gated OECTs were used for glucose detection, as carbon benefits from a high surface area and good electron transfer, which is desirable for enzymatic sensing^37^. The GOx enzyme solution in PBS was added to the well, and glucose was subsequently added at increasing concentrations with each injection. An example of a real-time detection experiment for glucose is shown in Fig. 5a, with the glucose injections indicated by arrows. The normalized response for three devices is shown in Fig. 5b. The relative response of the transistor is non-linear to the logarithm of the glucose concentration, as it has been observed in previous work^56^. In order to determine the limit of detection, a logarithmic regression is carried out in the concentration range from 1 μM to 1 mM, yielding a sensitivity of  − 3.4 ± 0.6%/dec (averaged over the three devices). The limit of detection is estimated as the noise level of the sensor divided by the sensitivity and multiplied by a confidence factor. Considering the standard deviation for the lowest concentration as the noise level and a confidence factor of 3, the limit of detection for glucose can be estimated to be about 5 μM, which is comparable to previous publications on non-transient OECT-based glucose sensors^6,10,57^. It can be seen in Fig. 5b that concentrations above 1 mM of glucose show higher device-to-device variations. This could be attributed to the larger amounts of H_2_O_2_ being released and affecting the PEDOT:PSS layer^58^, which would cause a permanent decrease in the current going through the channel. Therefore, the devices can detect glucose in a range from 5 μM to approximately 1 mM.

**Figure 6:**
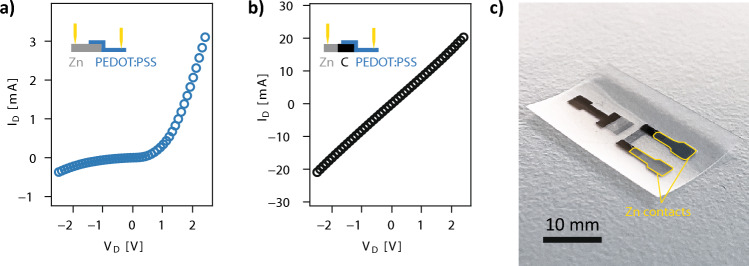
Integration of the OECTs with zinc metal traces. (**a**) I–V curve of a Zn-PEDOT:PSS junction. (**b**) I–V curve of a Zn-carbon-PEDOT:PSS junction. (**c**) Example device with carbon contacts for the channel and zinc interconnects.

Finally, we aimed to develop interconnects for the integration of the degradable OECTs with transient electronics. Indeed, the sensor may need to be connected to a readout circuit by conductive traces that are ideally eco- and/or bioresorbable. However, with the relatively high sheet resistance of the carbon ink (15 Ω/sq), the use of longer carbon traces may considerably increase the resistance of the system, leading to a reduction of the recorded currents and increased noise, as well as a shifting of the transconductance peak to higher gate voltages^46^. Metallic degradable and printable conductors are available and allow to reach higher conductivities^59^ and would be therefore suitable for the fabrication of interconnects in a fully printed degradable biosensing platform. The most investigated of such transient metals is zinc, for which methods have been developed^20,60^ to obtain conductivities superior to 10^5^ S/m. However, the direct integration of zinc as contacts for the OECTs presented here is challenging. Indeed, the zinc oxide layer that naturally forms in air on zinc metal forms a p–n junction with PEDOT, which is a p-type conductive polymer. As a result, a Schottky conduction regime emerges, with limited conductivity below a certain threshold^61,62^. The I–V characteristic of such a junction (formed by stencil-printed Zn and inkjet-printed PEDOT:PSS) is shown in Fig. 6a and displays a diode-like behavior. This type of contact is incompatible with the operation of the low-voltage OECTs presented here. However, zinc interconnects can be integrated with the carbon contacts used in the design presented above. The contact between carbon and PEDOT is ohmic, as shown by the linear I–V curve shown in Fig. 6b. As a consequence, zinc can be used to fabricate the interconnects of the OECT with carbon creating the contact with PEDOT:PSS. Such OECTs were fabricated and exhibit the same behavior as the carbon-based transistors presented before (Supplementary Fig. S3), demonstrating that the degradable OECTs can be integrated with high-conductivity biodegradable interconnects (Fig. 6c). This is of interest, as some transient applications may benefit from wireless operation for example, which is a promising direction for OECT-based devices^34^. It is noted that zinc degrades rapidly in aqueous solutions, and we have shown in a previous study that the zinc interconnects can be encapsulated in PLA and retain their electrical conductivity for several weeks in PBS^60^.

In this work, we demonstrate disposable and printed organic electrochemical transistors, which are suitable for the detection of ions and metabolites such as glucose. Their degradation in conditions analogous to industrial composting is observed over a period of one month. The disposable transistors are integrated with highly conductive biodegradable zinc interconnects on an eco- and bioresorbable substrate. The use of PLA as a substrate allows to manufacture sensors that are not only compatible with environmental applications, but can also be used for wearable and implantable applications. Concerning applications in bioresorbable electronics, it must be noted that despite PEDOT:PSS being biocompatible, the pathways and effects of its degradation in the body are not fully known and more investigations are needed on that topic. With an optimized design for the degradable transistors, the fabrication process is adjusted to be compatible with the use of polylactic acid as substrate. A silanization process allows the uniform deposition by inkjet-printing of the water-based PEDOT:PSS inks with good adhesion, enabling the fully additive manufacturing of the disposable transistors. The electrochemical transistor behavior is characterized and shown to be equivalent to non-degradable counterparts with similar design and channel characteristics. Carbon contacts are shown to be suitable for the fabrication of degradable OECTs, and the use of either a carbon or PEDOT:PSS gate to cause a shift in the transconductance peak while maintaining high transconductance values. Moreover, the use of non-polarizable gate materials is shown to decrease the dedoping behavior of the transistors, and a high gate area to be favorable. Sensitivities in the order of 10%/dec are observed for the detection of sodium, potassium and calcium ions. The ion sensing behavior of the devices is characterized with increasing and decreasing concentrations and is shown to be reproducible across devices. This indicates that the use of degradable materials and inks does not interfere with the sensing mechanisms in the measurement timescales compatible with point-of-care testing with disposable sensors. Although the sensing of several ions is demonstrated in this work, selective ion sensing would require a biocompatible and degradable ion-selective membrane (ISM), which would be an interesting direction for future developments. A proof-of-concept enzymatic detection of glucose is also shown, with linear behavior up to 1 mM. In order to ensure the applicability of the transistors as glucose sensors, more experiments should be conducted to characterize the stability and selectivity of the devices, ideally with the enzyme being immobilized on the sensor. The transistors are shown to be integrated with highly conductive degradable zinc tracks, which opens new possibilities for integration with biodegradable circuits, allowing for instance wireless operation.

## Experimental methods

### Substrate preparation and characterization

Polyimide foil (125 μm) was treated with oxygen plasma before printing the PEDOT:PSS channel to ensure good adhesion. Polylactic acid substrates were prepared from pellets (Ingeo™ Biopolymer 4032D). The PLA pellets were dissolved in 1,4-dioxane (Sigma Aldrich) at 50 °C overnight under stirring until obtention of a homogeneous viscous solution (15 wt%). The solution was blade cast with an automatic film applicator coater (Zehntner ZAA 2300) on a silicon wafer with a gap of 1000 μm and a speed of 2 mm/s. After drying overnight, films of a thickness of approximately 70 μm were obtained. The PLA film surface was activated with oxygen plasma and modified with a silanization process. 3-aminopropyl)triethoxysilane (APTES, Sigma Aldrich) was dissolved at 3 wt% in ethanol, spin-coated at 2000 rpm on the PLA films and allowed to cure at 80 °C for 20 mn, after which the excess APTES was rinsed with ethanol. The APTES layer was activated with oxygen plasma before PEDOT:PSS printing. The contact angles of the substrates with different surface treatments were measured with an optical contact angle measuring system (Dataphysics TBU 100) using deionized water as the liquid medium.

### OECT fabrication

A shellac-carbon paste was used for the source, drain and, in some experiments, gate contacts and its formulation was described previously^40^. The carbon paste was applied through a stencil mask made of one-sided polyethylene adhesive tape (Nexus G20, 80 µm). The mask was cut to the desired shape with a CO_2_ laser (Trotec Speedy300) and applied on the substrate, and the carbon paste was printed with the help of a silicone squeegee. After printing, the mask was peeled off and the carbon contacts were cured in an oven at 50 °C overnight. PEDOT:PSS printing solution was prepared by mixing 1.3 wt% PEDOT:PSS in water (Sigma Aldrich) with 5 wt% dimethyl sulfoxide (Sigma Aldrich) and 1 wt% (3-glycidyloxypropyl)trimethoxysilane. 4 layers of the solution were printed using a Dimatix DMP inkjet printer (Fujifilm) with a drop spacing of 30 μm and keeping the printer plate at 40 °C. The layers were then cured at 80 °C for 3 h or at 120 °C for 20 mn. The thicknesses of the contacts and channel were measured with a laser scanning confocal microscope (Keyence VK-X1000). Zinc interconnects were prepared by stencil printing a Zn microparticles (< 5 µm, Sigma Aldrich) mixed with pentanol (Sigma Aldrich) and polyvinylpyrrolidone (360 K, Sigma Aldrich). The zinc interconnects were sintered electrochemically to a conductivity of > 1000 S/cm as described previously^63^. They were then further sintered by photonic sintering with three 30 ms pulses (6.6 J/cm^2^)^60^. After OECT fabrication, the outlines of the devices were cut with a CO_2_ laser and the devices were manually peeled off from the wafer, as explained in a previous publication^60^. All subsequent measurements were done on the released devices.

### Test solutions preparation

1X phosphate-buffered saline (PBS) was prepared with chemicals sourced from Sigma Aldrich at the following concentrations in DI water: NaCl: 137 mM, KCl: 2.7 mM, Na_2_HPO_4_: 10 mM, KH_2_PO_4_: 1.8 mM. For potassium and sodium sensing tests, KCl and NaCl solutions were prepared in DI water. Glucose solutions were prepared by mixing glucose with PBS at the desired concentrations. Enzyme solutions were prepared before each experiment by mixing glucose oxidase (from Aspergillus Niger, 100 000 − 250 000 units/g, Sigma Aldrich) in 1 mL of PBS and letting them rest at room temperature for 30 min.

### Devices characterization

The devices were characterized with the test solutions confined in a laser-cut PMMA reservoir maintained on the device with acrylic adhesive, or for real-time measurements, by flowing the solution in a laser-cut PET and PDMS microfluidic channel^64^. For glucose measurements, the enzyme solution was first added to a PMMA well, followed by increasing concentrations of glucose solution. The source and drain contacts were isolated with polyurethane, which was cast from a 25% solution in DMSO, to avoid interaction between the carbon contacts and the electrolyte. The electrical measurements were performed using a semiconductor parameter analyzer (Agilent 4155A). Prior to measurements, the OECTs were rinsed with 1 ml DI water and left to stabilize in DI water under a gate-drain voltage of 600 mV for 5 min, repeated three times. Output curves (I_ds_–V_ds_) were measured with a medium integration time (20 ms) and varying the source-drain voltage between 0.1 and  − 0.9 V in steps of  − 20 mV and the gate-source between  − 1 and 1 V in steps of 200 mV. Transfer curves (I_ds_–V_s_) were measured with a long (320 ms) integration time and repeated 5 times to ensure stabilization of the device behavior, with V_ds_ set to  − 0.6 V and V_gs_ between  − 1 and 1 V with steps of 20 mV. The capacitance of the gates was measured by impedance spectroscopy (Metrohm Autolab 8 Series) as described previously^45^. The data were analyzed with custom software written in python.

## Supplementary Information

Supplementary Information 1.

## Data Availability

The data that support the findings of this work are available from the corresponding author upon reasonable request.
