# Supplementary material for: Organic electrochemical transistors printed from degradable materials as disposable biochemical sensors
Source: Sci Rep. 2023 Jul 15;13:11467. doi: 10.1038/s41598-023-38308-1 (PMC10349802; doi:10.1038/s41598-023-38308-1)
Supplement: Supplementary file 1 — Supplementary Information 1. [file 41598_2023_38308_MOESM1_ESM.pdf]

# Supplementary Information

## Organic Electrochemical Transistors Printed from Degradable Materials as Disposable Biochemical Sensors

N. Fumeaux*, C. P. Almeida, S. Demuru, D. Briand*

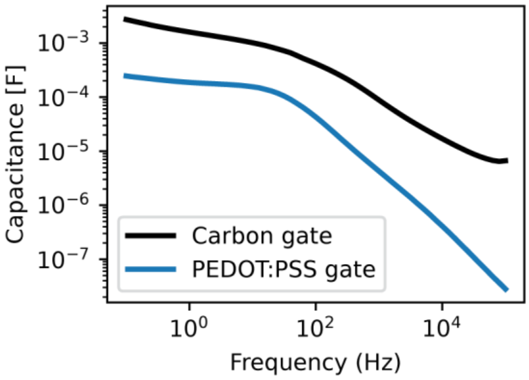


**Supplementary Figure 1**. Capacitance of the PEDOT:PSS and carbon gates measured in phosphate-buffered saline.

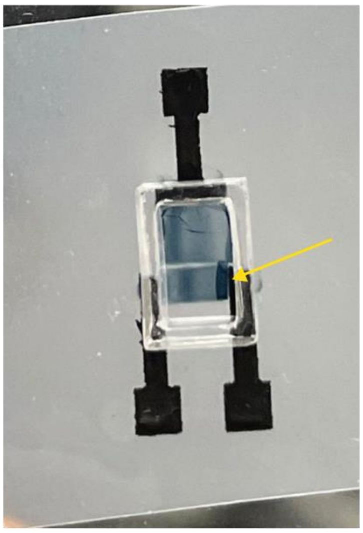

**Supplementary Figure 2**. Delamination (pointed out by the arrow) of PEDOT:PSS channel and gate in PBS solution, when inkjet printed on a polylactic acid film.

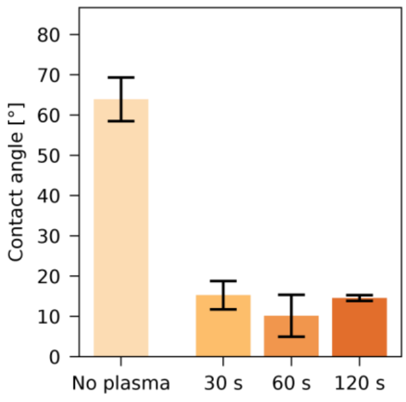


**Supplementary Figure 3**. Influence of oxygen plasma treatment on a polyimide film on the contact angle of a water droplet, with 40W, 40 kHz oxygen plasma treatments with variable time.

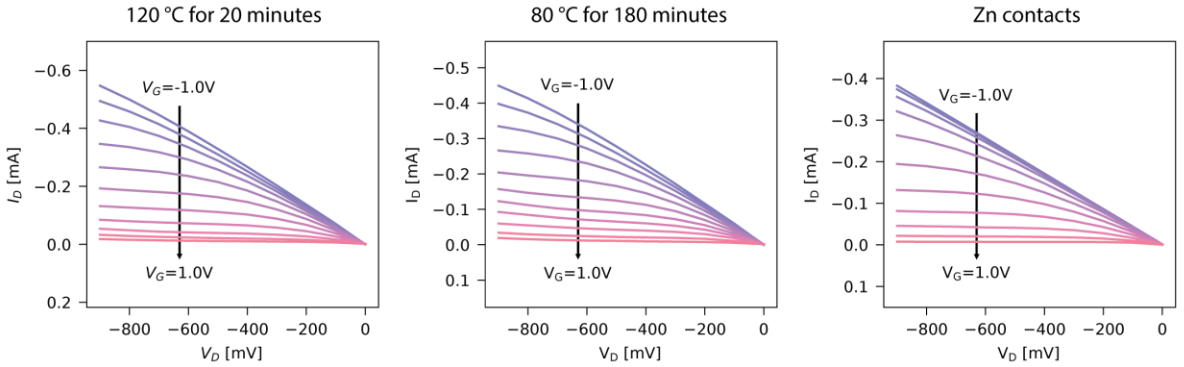


**Supplementary Figure 4**. Output characteristics of the OECTs (PEDOT:PSS-gated) for different modifications to the fabrication process (with the standard process as a control experiment on the left): changing the curing of the PEDOT:PSS layer to 80 °C for 3 hours (middle) and introducing Zn contacts using the same PEDOT curing protocol (right).

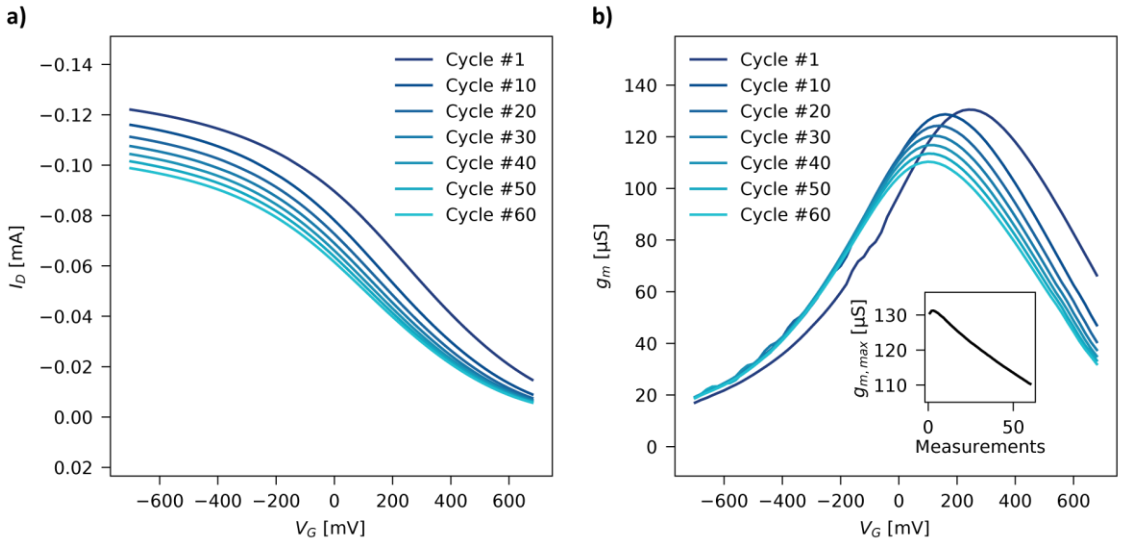

**Supplementary Figure 5**. Transfer characteristics of the OECTs on PLA after cycling transfer measurements for one hour. (a) Evolution of the transfer behavior after repeated measurements. (b) Evolution of the transconductance after repeated measurements, and the evolution of the maximal transconductance in the inset.

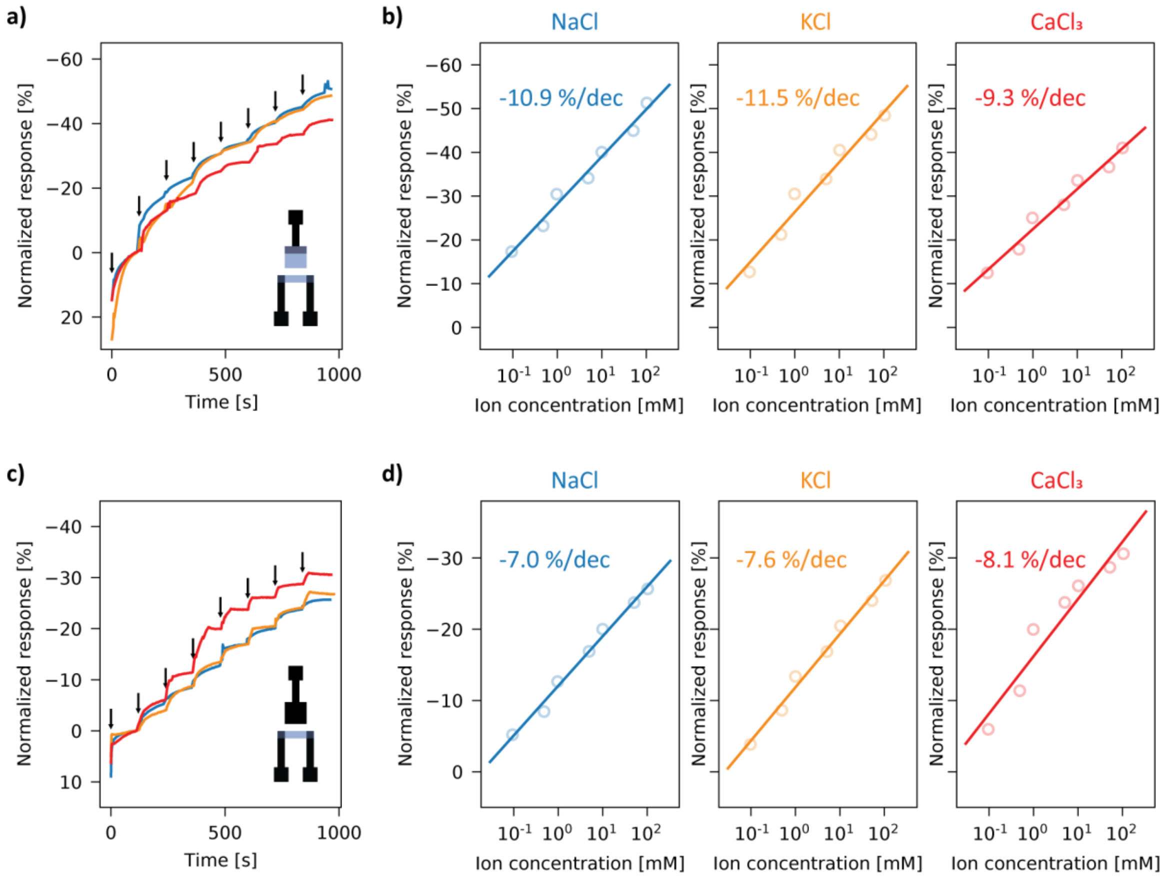


**Supplementary Figure 6**. Calibration curves for the OECTs for use in ion detection applications, for PEDOT:PSS-gated ((a) and (b)) and carbon-gated ((c) and (d)) devices. (a - c) Real-time normalized response to varying concentrations of ions, $Na^{+}$ (blue), $K^{+}$ (orange), $Ca^{3+}$ (red) with the injections of the different ion concentrations (0.1 mM, 0.5 mM, 1 mM, 5 mM, 10 mM, 50 mM, 100 mM) indicated by arrows. (b - d) Regression of the normalized current responses for each ion.

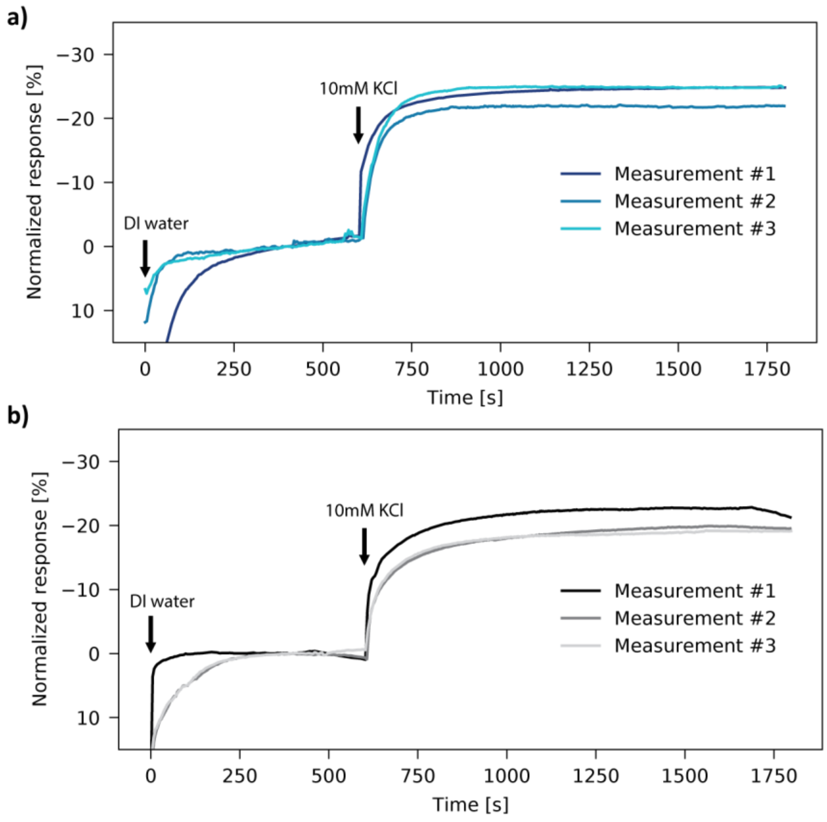


**Supplementary Figure 7**. Response dynamics and stability of the devices. Response of the devices after injection of 10 mM KCl injection, for 20 minutes, repeated three times, for (a) PEDOT:PSS-gated device and (b) carbon-gated device.
